# Misdiagnosis of HIV infection during a South African community-based survey: implications for rapid HIV testing

**DOI:** 10.7448/IAS.20.7.21753

**Published:** 2017-08-29

**Authors:** Tendesayi Kufa, Ayesha BM Kharsany, Cherie Cawood, David Khanyile, Lara Lewis, Anneke Grobler, Zawadi Chipeta, Alfred Bere, Mary Glenshaw, Adrian Puren

**Affiliations:** ^a^ Centre for HIV and STIs, National Institutes of Communicable Diseases, Sandringham, South Africa; ^b^ School of Public Health, University of the Witwatersrand, Johannesburg, South Africa; ^c^ Centre for the AIDS Programme of Research in South Africa (CAPRISA), Nelson R Mandela School of Medicine, University of KwaZulu-Natal, Durban, South Africa; ^d^ Research Department, Epicentre AIDS Risk Management (Pty) Limited, Paarl, Cape Town, South Africa; ^e^ Centres for Disease Control and Prevention (CDC), Pretoria, South Africa; ^f^ Division of Virology, School of Pathology, University of the Witwatersrand, Johannesburg, South Africa

**Keywords:** HIV, antibody testing, sensitivity, specificity, misdiagnosis

## Abstract

**Introduction**: We describe the overall accuracy and performance of a serial rapid HIV testing algorithm used in community-based HIV testing in the context of a population-based household survey conducted in two sub-districts of uMgungundlovu district, KwaZulu-Natal, South Africa, against reference fourth-generation HIV-1/2 antibody and p24 antigen combination immunoassays. We discuss implications of the findings on rapid HIV testing programmes.

**Methods**: Cross-sectional design: Following enrolment into the survey, questionnaires were administered to eligible and consenting participants in order to obtain demographic and HIV-related data. Peripheral blood samples were collected for HIV-related testing. Participants were offered community-based HIV testing in the home by trained field workers using a serial algorithm with two rapid diagnostic tests (RDTs) in series. In the laboratory, reference HIV testing was conducted using two fourth-generation immunoassays with all positives in the confirmatory test considered true positives. Accuracy, sensitivity, specificity, positive predictive value, negative predictive value and false-positive and false-negative rates were determined.

**Results**: Of 10,236 individuals enrolled in the survey, 3740 were tested in the home (median age 24 years (interquartile range 19–31 years), 42.1% males and HIV positivity on RDT algorithm 8.0%). From those tested, 3729 (99.7%) had a definitive RDT result as well as a laboratory immunoassay result. The overall accuracy of the RDT when compared to the fourth-generation immunoassays was 98.8% (95% confidence interval (CI) 98.5–99.2). The sensitivity, specificity, positive predictive value and negative predictive value were 91.1% (95% CI 87.5–93.7), 99.9% (95% CI 99.8–100), 99.3% (95% CI 97.4–99.8) and 99.1% (95% CI 98.8–99.4) respectively. The false-positive and false-negative rates were 0.06% (95% CI 0.01–0.24) and 8.9% (95% CI 6.3–12.53). Compared to true positives, false negatives were more likely to be recently infected on limited antigen avidity assay and to report antiretroviral therapy (ART) use.

**Conclusions**: The overall accuracy of the RDT algorithm was high. However, there were few false positives, and the sensitivity was lower than expected with high false negatives, despite implementation of quality assurance measures. False negatives were associated with recent (early) infection and ART exposure. The RDT algorithm was able to correctly identify the majority of HIV infections in community-based HIV testing. Messaging on the potential for false positives and false negatives should be included in these programmes.

## Introduction

HIV counselling and testing (HCT) is the gateway to care and treatment, including antiretroviral therapy (ART), for HIV-positive patients [1]. The widespread use of rapid diagnostic tests (RDTs) in high HIV prevalence settings has resulted in the increase in the number of people tested for HIV and the decentralization of HIV testing from health facilities into communities, reaching more young people, males, first-time testers and those at higher CD4 cell counts, and HIV-related less morbidity [2,3]. For example, 13.3 million people were tested for HIV in a national HIV testing campaign conducted in South Africa in the period 2011 to 2012 [4,5], and it is estimated that 9.9 million individuals were tested in 2015 [6]. With the introduction of the universal test and treat strategy and antiretroviral (ARV)-based HIV prevention strategies such as pre-exposure prophylaxis (PrEP), more and more people will be expected to test for HIV on a regular basis in order to initiate ART immediately or continue taking PrEP [7]. The need for HIV services to provide accurate HIV test results can therefore not be overstated.

HIV misdiagnosis occurs when an HIV-uninfected individual is incorrectly classified as HIV infected by the test used or vice versa [8]. There are multiple factors which can cause or contribute to HIV misdiagnosis. These vary from suboptimal testing strategies (including poor selection of assays used to construct algorithms and use of tiebreakers), deviation from standardized testing algorithms, user errors such as incorrectly performing test procedures, incorrectly interpreting test results, non-adherence to testing standard operating procedures as well as clerical errors [8–13]. False-positive rates as high as 10.3% upon retesting have been observed in some settings [14]. The consequences of HIV misdiagnosis are serious. False-positive HIV test results can result in the unnecessary treatment of HIV-uninfected individuals as well as exposure to the psychological trauma and stigmatization that may be associated with a diagnosis of HIV infection and the loss of credibility by HIV testing programmes [15]. On the other hand, false-negative HIV test results represent missed opportunities for entry into HIV care and treatment and the risk of unknowingly transmitting HIV to uninfected partners.

To reduce the risk of HIV misdiagnoses, the World Health Organization recommends the use of approved testing algorithms as well as the implementation HIV rapid test quality assurance programmes [8]. Key facets of these programs include training, retraining and mentoring of testing personnel, developing standardized registers to document HIV testing results, strengthening supply chains for RDT, developing standard operating procedures for rapid HIV testing, implementing internal and external quality controls, retesting and external quality assessments and proficiency testing as well as continuous monitoring and evaluation of these programmes [8]. These measures are essential especially for community-based testing programs where HIV testing may occur under less-than-ideal conditions with respect to the environmental temperatures at which RDT kits may be stored while in the field and the high volume of tests conducted. HIV testing in these settings is mostly conducted by community health workers who are generally well trained and highly proficient in HIV testing. However, in a few instances, lower accuracy has been documented among community workers compared to laboratory staff [16]. The performance of rapid HIV testing has also been found to vary with the reference standard used for evaluation. RDTs are second- or third-generation tests capable of detecting HIV-1 envelope protein antibodies, while fourth-generation tests are capable of detecting both antibodies to envelope proteins and p24 antigens [17–19]. The fourth-generation tests have been found to have fewer false positives and false negatives and should be better able to detect HIV infections earlier than third-generation tests [13]. We describe the overall accuracy and performance of the nationally recommended serial RDT algorithm against the nationally recommended laboratory-based fourth-generation immunoassays (IAs) in a household HIV prevalence survey during which rapid HIV testing was offered to willing and consenting participants. We discuss the implications of the findings for community-based HIV testing.

## Methods

### Study design and setting

Data used in this study were collected during a cross-sectional, household survey conducted in the Vulindlela and Greater Edendale sub-districts of uMgungundlovu district, KwaZulu-Natal, South Africa, during the period July 2015 to May 2016. This household survey was the second survey on the HIV Incidence Provincial Surveillance System (HIPSS) platform initiated in 2014 with the aim of establishing population-level estimates of HIV incidence and prevalence in the two sub-districts. The methods of the studies on this platform have been previously described [20]. Briefly, the HIPSS platform consists of two sequential cross-sectional household surveys conducted 1 year apart, each with approximately 10,000 individuals in the age group 15–49 years, residing in the Vulindlela and Greater Edendale sub-districts. Individuals were randomly selected from eligible households which in turn had been randomly selected from randomly selected census enumeration areas.

### Data collection procedures

Following eligibility assessment and informed consent procedures, eligible and willing individuals were enrolled into the second survey. A questionnaire was administered by trained field workers using personal digital assistants. Data on demographic, socio-economic and behavioural characteristics were collected as were data on access to HIV testing, care and treatment. Field workers then collected 25 ml whole blood specimens for HIV and related testing in the laboratory. Participants were offered field worker provided, rapid HIV testing in the home and referred to the local clinic for HIV care and treatment if the HIV result was positive. Field workers also completed a paper-based laboratory tracking form in which they documented rapid HIV test results in addition to other specimens collected.

### Rapid HIV testing and quality assurance

**Figure 1.**
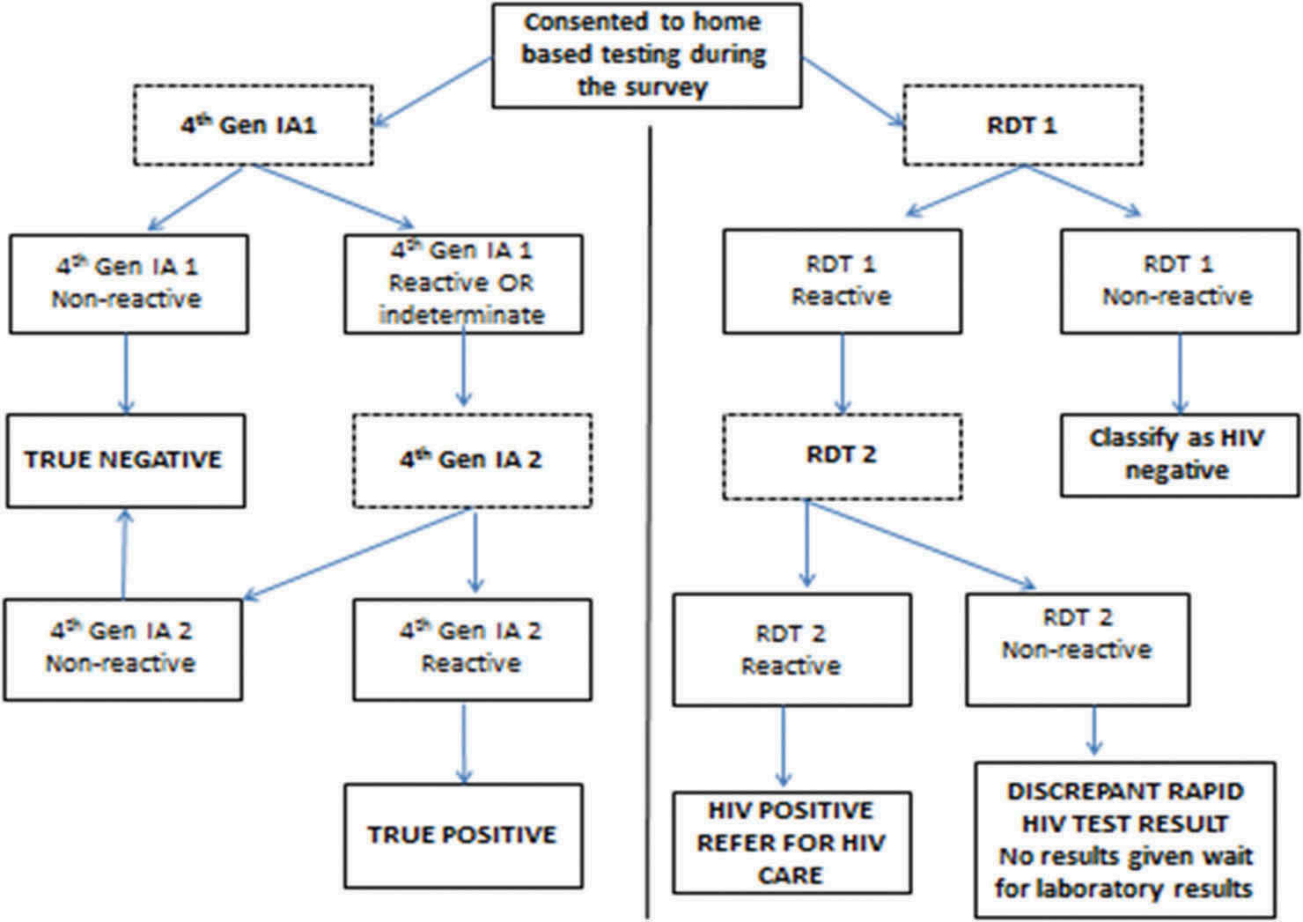
HIV testing algorithms. RDT 1 - Alere Determine HIV-1/2 (Matsudo, Japan), RDT 2 - UniGold HIV (Trinity Biotech, Bray, Ireland), 4th Gen IA1 - Vironostika HIV Uniform II antigen/antibody (Biomerieux, The Netherlands), 4th Gen IA2 - The Elecsys HIV Combi PT 4th Gen Assay, Roche Diagnostics, GmbH (Penzberg, Germany).

Figure 1 shows the HIV testing algorithms used for the community-based rapid testing and the reference fourth-generation IAs used in the laboratory. The rapid HIV testing algorithm used two RDTs in a serial algorithm: blood specimens collected by finger prick were tested with the first rapid test (RDT 1 - Alere Determine HIV-1/2, Matsudo, Japan), and if the test was reactive, a second rapid test (RDT 2 - UniGold HIV, Trinity Biotech, Bray, Ireland) was used to confirm the first HIV-reactive result. If RDT 2 was also reactive, the participant was considered HIV positive, received post-test counselling and referred to a local clinic for HIV care and treatment. If the RDT 1 was non-reactive, the participant was considered HIV negative and counselled on staying negative with and no further testing in the home. If the RDT 2 was non-reactive, the participant had a discrepant HIV result and was not given a result but was informed that the team will return with an HIV result once the laboratory testing was completed. The field workers were trained to conduct the rapid testing according to the manufacturer’s instructions, which for RDT 1 (Alere Determine HIV) meant collecting 50 µl of whole blood via finger prick, applying specimen to absorbent pad on the test strip, adding one drop of chase buffer and waiting 15 min (mechanically timed) before reading the result. For RDT2 (UniGold HIV), this meant applying two drops of blood specimen to the sample port, followed by two drops of wash reagent and waiting 10 min (also mechanically timed) before reading results. The algorithm used during the survey differed slightly to the nationally recommended algorithm [21], which at the time recommended the use of Advanced Quality Rapid anti-HIV [1,2] test (InTec Products INC) as a screening test with non-reactive results considered as HIV negative, and any reactive results confirmed with Abon HIV 1/2/0 Tri-Line Rapid test kit (Abon BioPharm). In the case of discrepant results, national algorithm recommended repeating the test with both the screening and confirmatory tests, and if still not resolved that, a blood specimen be collected for ELISA testing in the laboratory [21].

At appointment to the survey, the field workers who conducted the community-based HIV testing had an HCT certificate in accordance with National Department of Health guidelines. As part of the survey protocol training, they received an additional 2 days’ refresher training covering counselling and communication, national testing algorithms, rapid testing using survey-specific test kits, referrals and linkage into care and proficiency testing. Biweekly proficiency testing for the field workers was conducted throughout the survey with provision for retraining provided for those who failed it. The proficiency testing conducted at local field offices involved the laboratory sending specimen panels to the field workers to test and return results for comparison. Field supervisors also conducted random checks and shadowed home visits to ensure adherence to standard operating procedures.

### Laboratory HIV testing

Laboratory-based HIV testing used two fourth-generation IAs also in series. Participants’ blood specimens were first tested with the first IA (Vironostika HIV Uniform II antigen/antibody (Biomerieux, The Netherlands)), and if reactive (cutoff value = mean of three negative controls + 0.1), a second assay (The Elecsys® HIV Combi PT 4th Gen Assay, Roche Diagnostics, GmbH, Penzberg, Germany) was used to confirm the HIV reactive. The cutoff indices for the Elecsys® HIV Combi PT assay were as follows: 0.00–0.90 - non-reactive; 1:00–20 - weakly reactive/borderline; >20 - reactive. Following the manufacturer’s discontinuation of production of the Vironostika HIV Uniform II antigen/antibody assay, a small proportion of the samples (32 out of 10,236 tested for HIV in the laboratory (0.3%)) were tested using the Elecsys® HIV Combi PT 4th Gen Assay as the screening assay, and reactive results were confirmed using the Siemens Advia Centaur HIV Ag/Ab Combo (CHIV) assay (Siemens Healthcare Diagnostics Inc., Tarrytown, NY, USA). Cutoff index value of 1.0 was used to determine whether a specimen was reactive or non-reactive. All HIV-positive results were further confirmed by Western blotting and HIV viral load testing. Participants whose confirmatory (second) IA was reactive  were considered true positives while those whose initial screening test was non-reactive or  their  confirmatory  test was non-reactive, true negatives. Where the laboratory-based results and the community-based RDT results were discrepant, participants were informed of the laboratory-based results. In addition, if the RDT1 and RDT2 results were discrepant, participants were also informed of the laboratory-based results. Limiting antigen avidity enzyme immunosorbent assay (LAg Avidity EIA) testing was undertaken on all EIA antibody-positive samples to determine recent (early) HIV infection.

### Variables and outcomes

The main outcome of the study was the accuracy of the RDT algorithm, and this was defined as the overall proportion of individuals tested with an HIV RDT in the home who had the correct HIV result on the reference fourth-generation IA. Other outcomes determined were the sensitivity, specificity, positive predictive value (PPV), negative predictive value (NPV), false-positive rate and false-negative rate of the HIV RDT algorithm compared to the reference standard of laboratory-based IA algorithm. This reference standard was chosen to match the reference standard for HIV testing in the South African national HIV testing programme [21]. As the RDTs used in the survey were antibody-only HIV testing assays and were expected to be less sensitive compared to fourth-generation IA that detect for antibody and p24 antigen, their performance against an alternative reference standard, Western blot assay (New LAV Blot 11 Western blot assay, Bio-Rad, France), was also evaluated.

### Data analysis and statistical methods

The population tested by HIV RDT was described using descriptive statistics - median and interquartile ranges (IQR) for continuous variables as well as counts and proportions for categorical variables. The outcomes as described were determined as proportions with Wilson’s binomial 95% confidence intervals (CIs) calculated around the estimates. In order to assess any potential effects of immunological and virological status on the sensitivity of RDT, participants who tested false negative on RDT were compared to the true positives who were correctly diagnosed by RDT with respect to median CD4 cell count at enrolment, median viral load and proportion with viral loads >1000 copies/ml. The Wilcoxon rank sum and Chi-squared tests were used to assess statistically significant differences between these groups with *p*-values <0.05 considered statistically significant.

### Ethical considerations

The study was approved by the University of KwaZulu-Natal’s Biomedical Research Ethics Committee, the Centers for Disease Control and Prevention (CDC) Office of the Associate Directors of Science. Permissions to conduct the study were granted by the KwaZulu-Natal Provincial Department of Health and the uMgungundlovu District Municipality. Verbal informed consent was obtained from the head of the household and written informed consent obtained from the eligible individuals who were 18 years of age and older. For minors aged 15–18 years, written informed consent was obtained from the parents, guardians or caregivers and an individual assent obtained from the minor.

## Results

### Description of the community-based testing programme

A total of 50 field workers were trained and conducted the community-based rapid HIV testing. During the survey, none of the field workers failed proficiency testing, and there were no errors documented during proficiency testing or field supervision visits, although the documentation of proficiency testing and user errors was inconsistent and sometimes incomplete.

### Characteristics of participants included in the analysis

**Figure 2.**
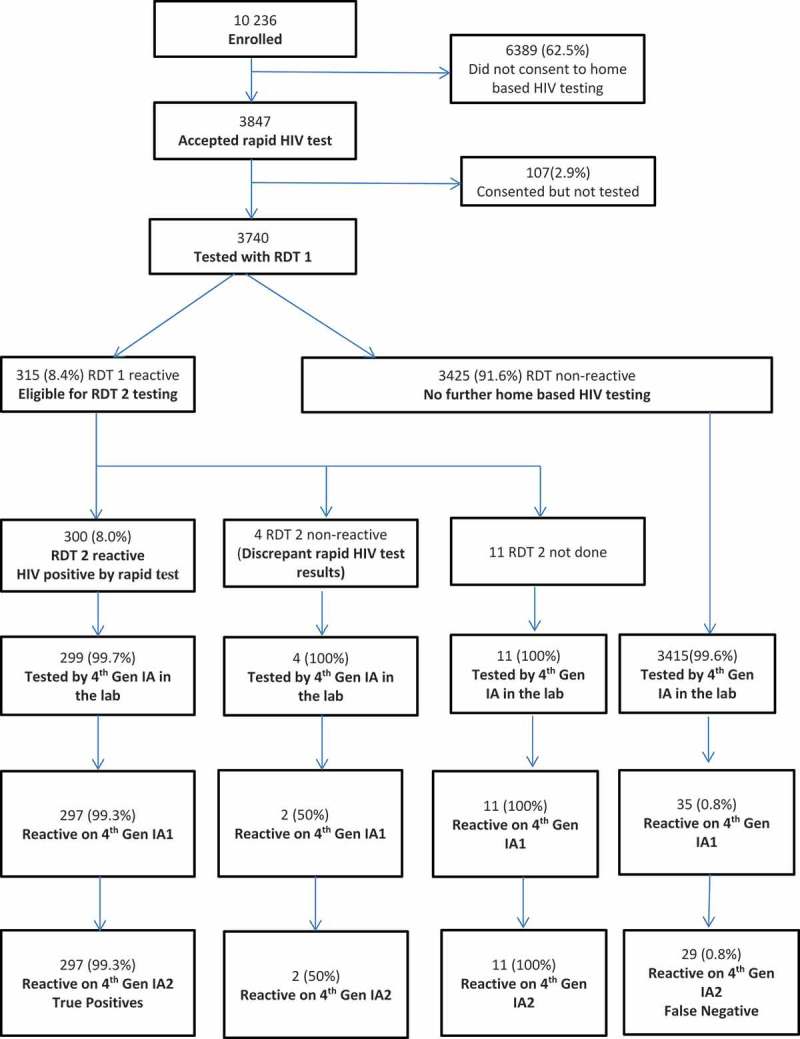
Study flow.

A total of 10,236 individuals were enrolled into the survey. Of these, 6389 (62.5%) did not consent to rapid HIV testing, while an additional 107 (1.0%) consented but ended up not testing. Of all the 6496 who were not tested, 5905 (90.9%) reported prior HIV testing, with 2749 (46.6%) self-reporting an HIV-positive status. The most common reason provided for declining an HIV test was prior knowledge of HIV status (4788 - 73.7%) and being afraid to test (1050 - 16.6%). In total, there were 3740 participants who were tested by RDT (see Figure 2). Table 1 shows the demographic and social characteristics of the participants who tested. The median age was 24 years (IQR 19–31 years), 1573 (42.1%) were males, 3092 (82.7%) were single, never been married or lived as married, and 3142 (84.0%) had been tested for HIV prior to the survey.

**Table 1.** Characteristics of participants who tested by rapid diagnostic test (RDT) in the home (*N* = 3740)

Variables	
Age (median, IQR) in years	24 (19–31)
Males (*n*, %)	1573 (42.1)
Black African (*n*, %)	3726 (99.6)
Completed 12 or more years of schooling (*n*, %)	1726 (46.1)
Single (never been married OR cohabited) (*n*, %)	3092 (82.7)
Perceived themselves to be at risk of HIV infection (*n*, %)	1436 (38.4)
Previous HIV testing (*n*, %)	3142 (84.0)
Self-reported being HIV positive	14 (0.5)
Self-reported taking ART at enrolment (*n*, %)	3 (0.1)
Tested HIV positive in the home	300 (8.0)
Final HIV-positive status^a^	339 (9.1)

^a^Out of 3729 as 11 tested by RDT in the home not tested by laboratory-based IA. IQR: interquartile range; ART: antiretroviral therapy.

**Table 2.** Performance of home-based rapid diagnostic test (RDT) compared to fourth-generation immunosorbent assay (EIA) and to fourth-generation EIA and Western blot (*N* = 3708)

	Fourth-generation immunoassay		Fourth-generation immunoassay and Western blot	
Parameters	*n*/*N*	% (95% CI)	*n*/*N*	% (95% CI)
Accuracy	3677/3729	98.8 (98.5–99.2)	3680/3708	99.2 (98.1–99.5)
Sensitivity	297/326	91.1 (87.5–93.7)	297/323	92 (88.5–94.4)
Specificity	3380/3382	99.9 (99.8–100)	3383/3385	99.9 (99.8–100)
Positive predictive value	297/299	99.3 (97.6–99.8)	297/299	99.3 (97.6–99.8)
Negative predictive value	3380/3409	99.1 (98.8–99.4)	3383/3409	99.2 (98.9–99.5)
False-positive rate	2/3382	0.06 (0.02–0.22)	2/3385	0.06 (0.02–0.22)
False-negative rate	29/326	8.9 (6.3–12.5)	26/323	8.0 (5.6–11.5)

RDT 1 - Alere Determine HIV-1/2 (Matsudo, Japan); RDT 2 - UniGold HIV (Trinity Biotech, Bray, Ireland), 4th Gen IA1-Vironostika HIV Uniform II antigen/antibody (Biomerieux, The Netherlands), 4th Gen IA2 - The Elecsys® HIV Combi PT 4th Gen Assay, Roche Diagnostics, GmbH (Penzberg, Germany), Western blot assay - New LAV Blot 11 Western blot assay (Bio-Rad, France). CI: confidence interval.

**Table 3.** Immunological and virological profiles of false negatives compared to true positives

Variables	HIV positive (false negatives) (*N* = 29)	HIV positive (true positives) (*N* = 297)	*p*-Value
Detectable viral load (viral load >20 copies/ml) (*n*, %)	26 (89.7)	267 (89.9)	0.967^a^
HIV RNA viral load, copies/ml (median, IQR)	17,000 (5600–54,000)	24,000 (3700–100,000)	0.679^c^
Viral load >1000 copies/ml, (*n*, %)	23 (79.3)	246 (82.8)	0.634^a^
CD4 cell count (median, IQR) cells/µl	557 (200–753)	430 (266–610)	0.308^c^
CD8 cell count (median, IQR) cells/µl	880 (750–1346)	990 (776–1343)	0.487^c^
CD4:CD8 ratio (median, IQR)	0.5 (0.2–0.9)	0.4 (0.2–0.6)	0.2^c^
LAg avidity EIA positive (*n*, %)	8 (27.6)	22 (7.4)	0.001^b^
Western blot negative or indeterminate (*n*, %)	3 (10.4)	0 (0.0)	0.001^a^
Acute infection (*n*, %)	2 (6.9)	0 (0.0)	0.001^a^
Self-reported being HIV positive (*n*, %)	3 (10.3)	8 (2.7)	0.064^a^
Self-reported taking ART (*n*, %)	1 (3.5)	1 (0.3)	0.170^a^

^a^Fisher’s exact Chi-squared test. ^b^Chi-squared test. ^c^Wilcoxon rank sum test *p*-value. LAg avidity EIA: limiting antigen avidity enzyme immunosorbent assay; IQR: interquartile range; ART: antiretroviral therapy.

### Rapid HIV testing results

Of the 3740 participants tested with RDT 1, 315 (8.4%) were reactive and were eligible for testing using RDT 2. Of these, 11 were not tested with RDT 2 (reasons not provided), 300 were reactive on RDT 2 and 4 were non-reactive and therefore had discrepant RDT results (Figure 2). The prevalence of discrepant rapid HIV test results was 0.1% among all those tested by RDT and 1.3% among those who tested positive on RDT 1. Of the four with discrepant results, two (50%) were subsequently confirmed to be HIV positive with the laboratory IA testing algorithm.

### Performance of the RDT compared to laboratory-based IA tests

Among the 3740 participants tested using the RDT algorithm, there were 3708 (99.1%) who had a definitive result on the RDT algorithm and were tested for HIV using the fourth-generation IA algorithm in the laboratory; excluding 11 not tested by RDT, 4 with discrepant RDT results and 11 who were not tested by fourth-generation IA in the laboratory (1 RDT-positive and 10 RDT-negative reasons not stated) and 6 who had discrepant EIA results (Figure 2). Of the 3708, 326 (8.8%) were reactive on the laboratory fourth-generation EIA algorithm and therefore considered the true HIV positives, while 3382 (91.2%) were non-reactive on the laboratory fourth-generation EIA and considered true HIV negatives. Of the true negatives, two (0.06%; 95% CI 0.02–0.22) false positives were identified. Among the 326 true HIV positives, 29 (8.9%; 95% CI 6.3–12.5) had tested HIV negative by RDT algorithm at home and were therefore false negatives (see Table 2). The overall accuracy of the RDT algorithm in the home-based testing was 98.8% (95% CI 98.5–99.2). However, the sensitivity of the RDT algorithm in this setting was lower than expected at 91.1% (95% CI 87.5–93.7). The specificity of the RDT algorithm was 99.9% (95% CI 99.8–100). The positive and negative predictive values were 99.3% (95% CI 97.6–99.8) and 99.1% (95% CI 98.8–99.4). When compared against the fourth-generation EIA as a reference standard and Western blot (which identified 323 HIV-positive individuals compared to 326 using fourth-generation testing alone), the sensitivity, specificity, PPV and NPV of RDTs were 92% (95% CI 88.5–94.4), 99.9% (95% CI 99.8–100), 99.3% (95% CI 97.4–99.8) and 99.2% (95% CI 98.9–99.5) respectively. The false positives and false negatives were 0.06% (95% CI 0.02–0.22) and 8.0% (95% CI 5.6–11.5) respectively.

In an analysis excluding 13 individuals (*N* = 3695) who self-reported being HIV positive and were tested by both RDT in the home and EIA in the lab, the sensitivity, specificity, PPV and NPV of RDTs were 91.7% (95% CI 88.1–94.3), 99.9% (95% CI 99.8–100), 99.3% (95% CI 97.3–99.8) and 99.2% (95% CI 98.9–99.5), respectively. The false-positive and false-negative rates unchanged at 0.06% (95% CI 0.02–0.24) and 8.3% (95% CI 5.7–11.9), respectively.

### Description of the false positives

There were two individuals who were falsely positive on RDT. Both individuals were female, were not pregnant, were not taking any medications and did not suffer from chronic illnesses at the time of enrolment. These two individuals both reported testing HIV negative within the preceding 90 days. There were no obvious reasons to explain the false-positive results. However, clerical errors cannot be excluded.

### Comparison of virological and immunological profiles of false negatives to true positives

HIV-positive individuals who were incorrectly diagnosed as HIV negative by the RDT (false negatives) were not significantly different from those who were correctly diagnosed as HIV positive by RDT with respect to proportions who had detectable HIV RNA and median viral load (see Table 3). There was also no difference in the median CD4 counts (557 cells/µl (IQR 200–753 cells/µl)) vs. 430 cells/µl (266–610 cells/µl), *p* = 0.380) and median CD4: CD8 ratios (0.5 (95% CI 0.2–0.9) vs. 0.4 (95% CI 0.2–0.6), *p*-value 0.2) among the false negatives. False-negative individuals were also more likely to be LAg avidity EIA positive (27.6% vs. 7.4%, *p* = 0.001), to report being HIV positive (10.3% vs. 2.7%, *p* = 0.006) and taking ART at enrolment (3.5% vs. 0.3%, *p* = 0.170) although the latter represented only one individual in each group. Among the false negatives were two individuals who met criteria for acute HIV infection (Western blot negative or indeterminate AND detectable viral load) accounting for only 0.6% of all true HIV positives and 6.9% of the false negatives.

## Discussion

We describe the performance of RDT in a serial algorithm used for community-based HIV testing during a household survey to measure HIV prevalence and incidence. In this setting, the overall accuracy of the RDT algorithm compared to a reference standard of fourth-generation laboratory-based IAs was high at 99.0%, but sensitivity was lower than the WHO-recommended level of ≥99% at 91.1% with a false-negative rate of 8.9%. Participants incorrectly diagnosed as HIV negative by the RDT algorithm did not differ significantly from those correctly diagnosed as HIV positive with respect to CD4 cell counts, CD8 cell counts, CD4:CD8 ratios and median viral loads among those with detectable virus, although were more likely to be classified as recently infected by the LAg assay and to self-report being HIV positive. There was a low false-positive rate at 0.06%. The performance of the RDT was similar when comparing the fourth-generation IAs and Western blot (equivalent to the third-generation HIV testing) as reference methods.

The low sensitivity of the RDT in community-based testing was unexpected and concerning. Several studies of RDT performance in both community- and health facility-based HIV testing have reported higher sensitivities than observed in our study. Molesworth et al. found a sensitivity of 99% comparing third-generation RDT kits with third-generation EIA during HIV testing in the context of a household survey, using initially two RDTs in parallel with a tiebreaker, then two RDTs in series with a tiebreaker and finally retesting all positives and 10% of the negatives in the laboratory [22]. Jackson et al. found a sensitivity of 98% in a community randomized controlled trial of home-based HCT conducted by lay counsellors again comparing third-generation RDT kits with a third-generation EIA [23]. Wolpaw et al. found an RDT sensitivity of 68.7% which improved to 93.5% following switching test kit brands and to 98% following implementation of quality improvement measures upon retesting individuals who previously tested HIV negative at primary care clinics in Cape Town, South Africa [24]. In this Cape Town study, the inconsistent use of chase buffer and early reading of results were common errors observed and targeted for quality improvement interventions [24].

This reduced sensitivity has wide-ranging implications for HIV prevention, care and treatment in South Africa. A false-negative result may result in inadvertent transmission of HIV to uninfected partners by individuals who believe they are HIV negative. With the rollout of PrEP among men-who-have-sex-with-men and female sex workers [25], a false-negative diagnosis implies continuing with PrEP when a full treatment regimen is required which may lead to ARV drug resistance. With the implementation of universal test and treat, a false-negative diagnosis may also result in delayed entry into care, resulting in excess morbidity and mortality from HIV.

A number of factors could explain the lower sensitivity of the RDT algorithm observed in our survey. The use of fourth-generation EIA has been found to have fewer false positives and false negatives and able to detect more acute infections compared to third-generation tests [13]. However, the relatively low proportion of false-negative individuals who had acute HIV infection (10.4% of true positives and 0.09% of RDT negatives) suggests that this was unlikely to be a main factor contributing to the high false-negative rate. These rates of acute infections observed in our study were comparable to rates reported elsewhere in the country [26]. In addition, limiting the analysis of performance to a reference standard of fourth-generation EIA and Western blot (equivalent to third-generation HIV testing) did not change the performance of the rapid testing. There may have been undocumented user errors during home-based testing with RDT despite the implementation of a quality assurance programme including a proficiency testing. Another factor contributing to the reduced sensitivity observed could have been the selection and sequence of RDT kits used in the survey’s serial testing algorithm. The algorithm had Determine as a screening test and UniGold as confirmatory. Studies of laboratory-based comparisons of RDT test kit performance have demonstrated lower than expected sensitivities with both Determine and UniGold test kits in certain settings. Gawalingo et al. reported a sensitivity of 97.3% for a serial algorithm which used Determine as the screening test [27]. Kosack et al. demonstrated a sensitivity of 96.2% for UniGold in a head-to-head comparison of eight RDT kits in the laboratory [28]. Because survey specimens were not tested by any other RDT in the laboratory, the contribution of RDT selection to the reduced sensitivity observed cannot be ruled out. In our study, there may have additional population-level factors such as gender and geographical location as well as others yet to be determined which may have affected RDT performance, as also reported by Kosack et al. [28]. Lastly, individuals who were false negative may have been recently infected with detectable viral loads and higher CD4 cell counts. This is supported by the association between false-negative HIV results with positive LAg avidity assay which may indicate recent infection although false recency can occur [29]. However, the LAg assay is designed to detect infections up to 6 months in duration, a duration by which most HIV tests should be able to detect sufficient antibodies in the blood. Falsely negative individuals in the study could also have been on long-term ART. A few studies have documented low antibody titres and even seroreversion with long-term ART initiated during acute or early infection and continued long term with sustained viral suppression. Early ART initiation has also been associated with delayed antibody maturation following infection leading false recency of infection [30,31]. Retesting individuals on ART is currently not recommended [8].

Our analysis presents the performance of RDT during community-based household HIV testing. The analysis included a large group of randomly selected individuals giving relatively precise estimates of rapid HIV test performance and minimizing selection bias. Enrolling and testing one individual per household likely minimized clerical errors related to mixing-up participant results or specimens. In addition, there was laboratory confirmation of HIV status for all tested in the home, allowing direct comparison of RDT test performance. However, only participants who wanted home testing were tested, thus bringing bias in. For example, because of this self-selection, 14 participants who had self-reported being HIV positive including three reporting current or past ART use were also enrolled. However, a sensitivity analysis excluding these participants produced similar RDT performance.

Our study had a few limitations. Previous HIV testing and ART use were self-reported and not verified in the laboratory, so we were unable to determine the effect of retesting and ART use on test performance. Although a few individuals self-reported taking ART at enrolment (*n* = 3), it would have been ideal to validate this by testing plasma ARV levels in the laboratory. Although proficiency testing was conducted biweekly, there was incomplete and inconsistent documentation of user errors, storage and environmental conditions under which test kits were stored or used, all of which can affect RDT performance in the field. Lastly, the use of a less specific reference standard, two fourth-generation IAs, despite availability of more specific tests was another limitation of this analysis. This reference standard was used in order to mirror the reference standard used for resolving discrepant results in the national HIV testing programme. The use of fourth-generation testing with Western blot which showed comparable performance showed that this was not a major limitation. Despite these limitations, our study provides valuable information and lessons on the performance of RDT in home-based testing settings. Whether the lower than expected sensitivity observed in this study is due to the test or operator performance, the need to strengthen systems for correct storage of test kits and quality assurance programmes and using the results thereof to improve quality cannot be understated.

In conclusion, our study showed high accuracy using the RDT algorithm and the potential for the large-scale roll-out of community-based testing. However, reduced sensitivity with higher than expected false negatives associated with recent infection was observed. As the RDT algorithm showed high accuracy and ability to reliably identify the majority of HIV infections, its use in community-based HIV testing programmes should be promoted and scaled up as it reaches more people. However, messaging on the potential for false positives and false negatives should be included in HIV testing programmes and nucleic acid amplification testing considered for those on PrEP. In addition, the national HIV testing programme should regularly monitor and validate the rapid HIV testing algorithms and revised these as guided by the findings.
